# Supplementary material for: The Antitumor Agent Ansamitocin P-3 Binds to Cell Division Protein FtsZ in Actinosynnema pretiosum
Source: Biomolecules. 2020 Apr 30;10(5):699. doi: 10.3390/biom10050699 (PMC7277737; doi:10.3390/biom10050699)
Supplement: Supplementary file 1 [file biomolecules-10-00699-s001.pdf]

biomolecules

MDPI

Article

The Antitumor Agent Ansamitocin P-3 Binds to Cell Division Protein FtsZ in *Actinosynnema pretiosum*

Xinran Wang[1,2], Rufan Wang[1], Qianjin Kang[1,2] and Linquan Bai[1,2,*]

[1] State Key Laboratory of Microbial Metabolism, School of Life Sciences & Biotechnology, Shanghai Jiao Tong University, Shanghai 200240, China
[2] Joint International Research Laboratory of Metabolic & Developmental Sciences, Shanghai Jiao Tong University, Shanghai 200240, China
* Correspondence: bailq@sjtu.edu.cn

Table S1. Strains, plasmids and primers used in this study.

Strains or plasmids	Features	Sources
Actinosynnema pretiosum		
NXJ-24	ATCC 31280Δ*ansa30* pSET152::*kasOp*-asm10*	[1]
WXR-24	ATCC 31280Δ*ansa30*Δ*mec-pks7*::*kasOp*-asm10*	This study
WXR-30	WXR-24 pLQ1913	This study
E. coli		
DH10B	F^- (*tra*Δ*36 proAB lacIq lacZ*Δ*M15*) *rpsL* (*strR*) *thr leu endA thi-1 lacY galK galT ara tonA tsx dam dcm supE44* Δ(*lac-proAB*) Δ(*mcrC-mrr*)102::Tn10 (*tetR*)	GIBCO-BRL
ET12567(pUZ8002)	*recE, dam, dcm, hsds, cm, str, tet, km*	[2]
**Plasmids**		
pET28a	*pBR322 ori, kan, lacI,* P_{T7}	Novagen
pSET152	*ΦC31 int, aac(3)IV lacZa* $oriT_{RK2}$	[2]
pLQ646	pSET152::*kasOp**	This study
pLQ586	pSET152::*kasOp*-asm10*	[1]
pLQ1913	pLQ646::*APASM_5716*	This study
pLQ1914	pET28a:: *APASM_5716*	This study
**Primers [a]**	**Sequence (5′ – 3′) [b]**	
5716-646-FP	GAAGAGGTGACGTCCATATGACCCGGTGGTCATCCACTC	
5716-646-RP	TATGACATGATTACGAATTCTCAGCGCCGCATGAACGG	
5716-28a-FP	ATTACATATGACCCGGTGGTCATCCACTC	
5716-28a-RP	ATATGAATTCTCAGCGCCGCATGAACGG	

[a] FP stands for forward primer, and RP stands for reverse primer. [b] The underlined nucleotides of 5716-28a-FP and 5716-28a-RP refer to *Nde*I and *Eco*RI restriction sites used for the cloning with pET280a; while the underlined nucleotides of 5716-646-FP and 5716-646-RP represent the overlapping regions in adjacent to the *Nde*I and *Eco*RI sites of pLQ646, which were used for one-step assembly of the amplified fragment with *Nde*I and *Eco*RI treated pLQ646.

Table S2. CFUs and cell survive rates (%) of WXR-24 and WXR-30 counted after 3 days on YMG plates containing different concentrations of AP-3.

**Strains**	**AP-3 concentrations (mg/L)**				
	**0**	**100**	**200**	**300**	**400**
**WXR-24**	2,053 ± 360 /100	2,052 ± 234 /100 ± 11	1,485 ± 31 /72 ± 2	0/0	0/0
**WXR-30**	2,350 ± 221 /100	2,410 ± 153 /100 ± 7	2,242 ± 169 /95 ± 7	15 ± 4 /0.63 ± 0.17	8 ± 5 /0.34 ±0.21

Table S3. Average colony sizes (× 10^{-2} mm^2) of WXR-24 and WXR-30 on YMG plates containing different concentrations of AP-3.

**Strains**	**AP-3 concentrations (mg/L)**		
	**0**	**100**	**200**
**WXR-24**	6.08 ± 4.24	3.74 ± 3.07	1.27 ± 1.33
**WXR-30**	7.19 ± 4.39	4.95 ± 3.58	3.05 ± 3.67

Table S4. Binding constants of ansamitocin P-3 to FtsZ.

**Protein**	**Chemical**	**Binding model**	***K*a ($M^{-1}s^{-1}$)**	***K*d (s^{-1})**	**K_D (M)**
FtsZ	AP-3	1:1 binding	$2.36{\times}10^3$	0.794	$3.36{\times}10^{-4}$

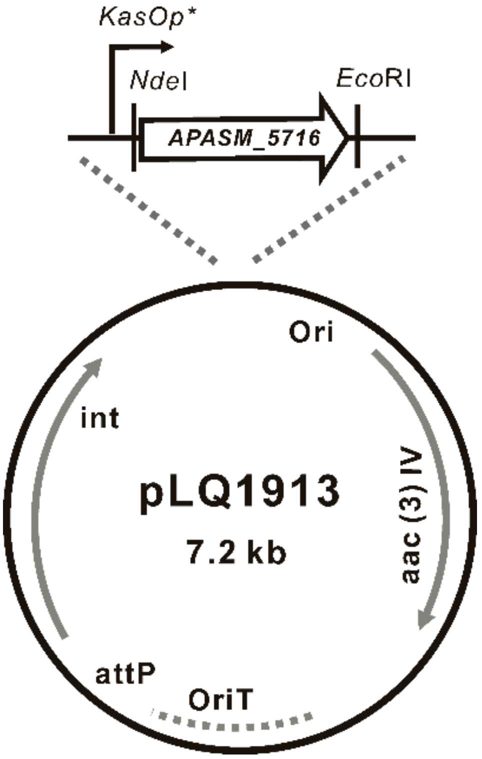

Figure S1. Schematic representation of *APASM_5716* overexpression. *APASM_5716* was inserted into the *Nde*I/*Eco*RI sites of vector pLQ646 under the control of strong promoter *kasOp**.

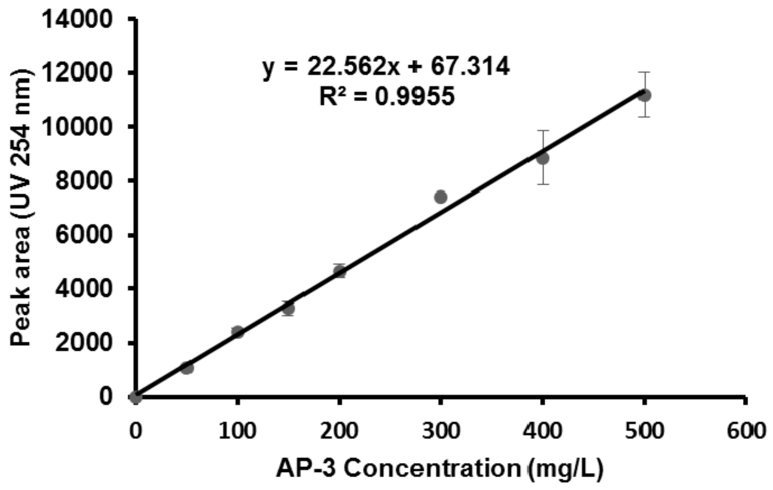

Figure S2 HPLC standard curve of AP-3. AP-3 solutions of 0, 50, 100, 150, 200, 300, 400, 500 mg/L were analyzed by HPLC to obtain the corresponding peak areas.

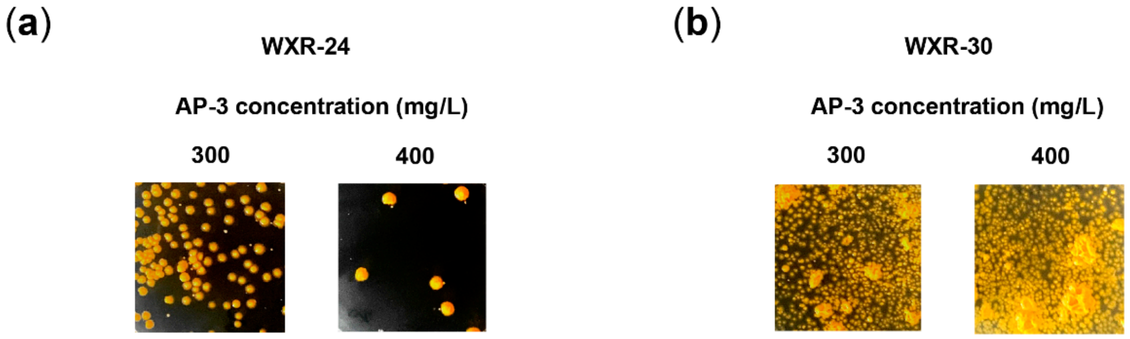

Figure S3. Growth of *A. pretiosum* WXR-24 (a) and WXR-30 (b) on YMG plates containing 300 mg/L or 400 mg/L AP-3. The photos were taken on day 16 after inoculation.

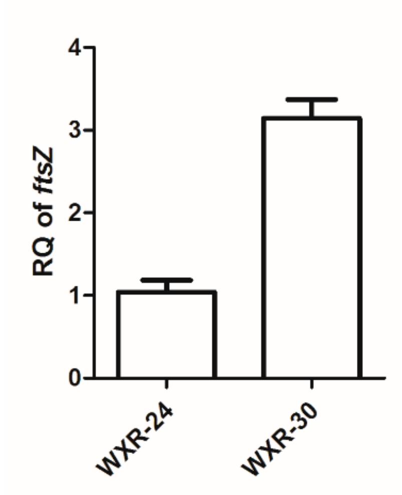

Figure S4. Transcription level of *ftsZ* in WXR-24 and WXR-30. The Y-axis scale stands for the expression of *ftsZ* relative to that of *hrdB*. The average expression value of *ftsZ* in the control strain WXR-24 is set to 1 as the standard, and the expression values of WXR-30 are accordingly presented.

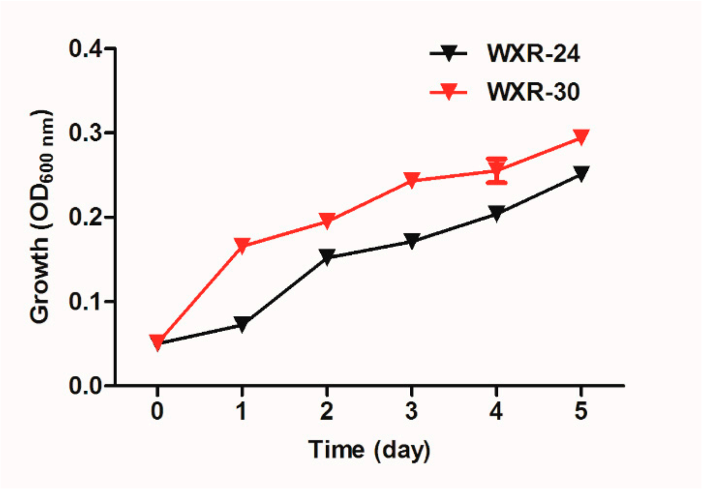

Figure S5. Growth of *A. pretiosum* WXR-24 and WXR-30 in the fermentation broth. Mean values of three independent experiments with SD are indicated by error bars.

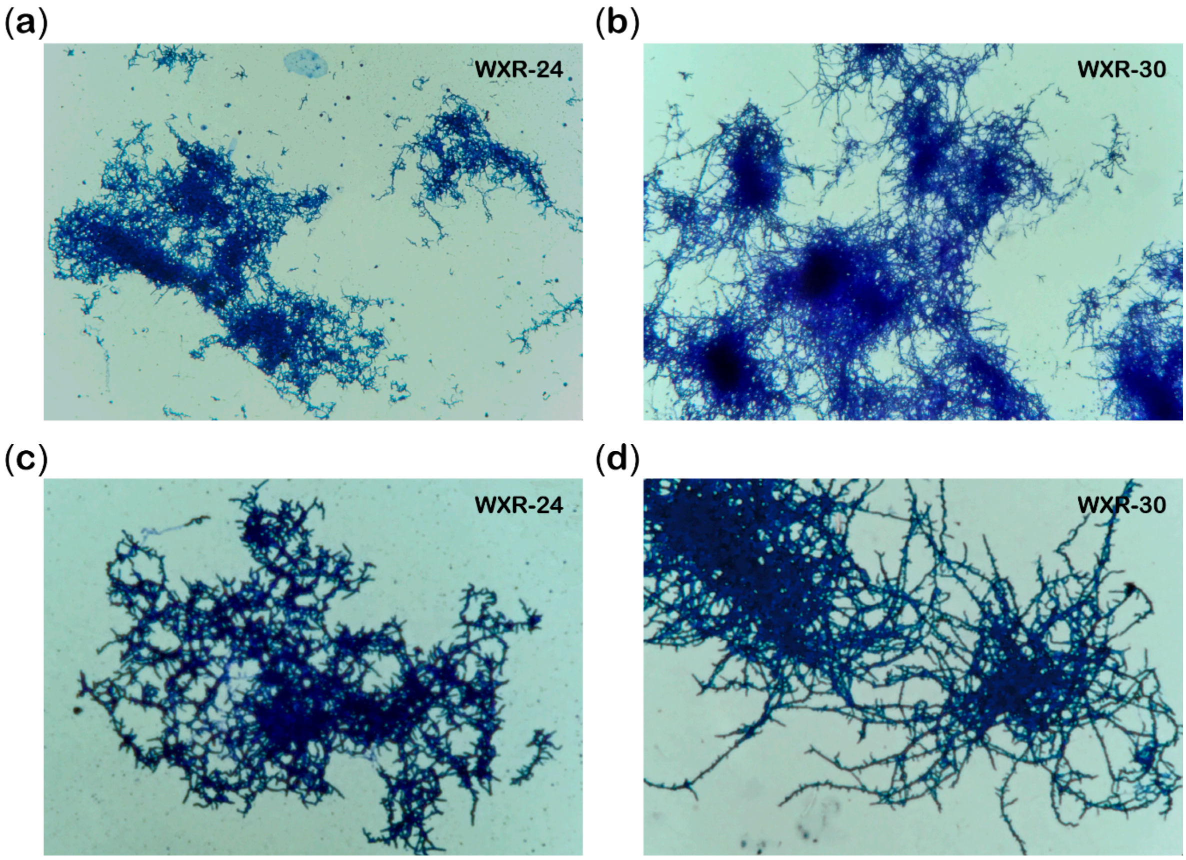

Figure S6. Mycelial morphology of WXR-24 (a and c) and WXR-30 (b and d) on the first day of fermentation, observed by optical microscope under 20x objective (a and b) and 60x objective (c and d). The mycelia were stained by the crystal violet staining solution.

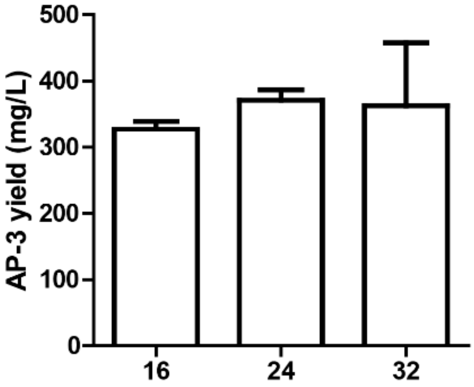

Figure S7. AP-3 yields of WXR-30 in fermentation media supplemented with 16 g/L, 24 g/L or 32 g/L yeast extract. Mean values of three independent experiments with SD are indicated by error bars.

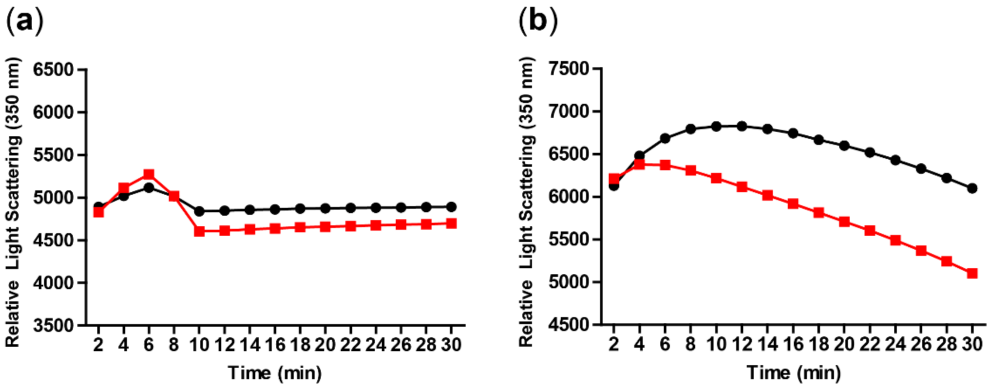

Figure S8. The inhibition of FtsZ assembly by AP-3 with FtsZ from *Streptomyces coelicolor* (a) and *Bacillus subtilis* (b) as detected by the real-time light scattering assay. Fluorescence signals of the reaction sample with (red line) or without (black line) 500 µM AP-3 were recorded every 2 min.

References

1. Ning, X.; Wang, X.; Wu, Y.; Kang, Q.; Bai, L. Identification and engineering of post-pks modification bottlenecks for ansamitocin P-3 titer improvement in *Actinosynnema pretiosum* subsp. *pretiosum* ATCC 31280. *Biotechnol. J.* **2017**, *12*, doi:10.1002/biot.201700484.
2. Kieser, T.; Bibb, M.J.; Butter, M.J.; Chater, K.F.; Hopwood, D.A. *Practical Streptomyces Genetics*, John Innes Centre: Norwich, England, **2000**.

© 2020 by the authors. Licensee MDPI, Basel, Switzerland. This article is an open access article distributed under the terms and conditions of the Creative Commons Attribution (CC BY) license (http://creativecommons.org/licenses/by/4.0/).
